# Characterization of the DNA molecular sequence of complete plastid genome of *Paeonia rockii* subsp. *taibaishanica*, an endemic species in China

**DOI:** 10.1080/23802359.2021.1917311

**Published:** 2021-08-13

**Authors:** Peng-Bin Dong, Li Zhang, Zhao-Ping Lu, Yuan Wang, Xiao-Yu Song, Jiu-Xia Wang, Dan He, Xiao-Feng Lei, Ma-Li Wang, Min-Feng Fang, Zhong-Hu Li

**Affiliations:** a Key Laboratory of Resource Biology and Biotechnology in Western China, Ministry of Education, College of Life Sciences, Northwest University, Xi’an, PR China; b State Key Laboratory of Grassland Agro-ecosystems, School of Life Sciences, Lanzhou University, Lanzhou, PR China

**Keywords:** Chloroplast genome, *Paeonia rockii* subsp. *taibaishanica*, phylogenetic tree

## Abstract

*Paeonia rockii* subsp. *taibaishanica* (Paeoniaceae), one of the tree peony species, is endemic to the Qinling Mountains in central China. In this study, we characterized its whole plastid genome sequence using the Illumina sequencing platform. The complete plastid genome size of *P. rockii* subsp. *taibaishanica* is 153,368 bp in length, including a large single copy (LSC) region of 85,030 bp, a small single copy (SSC) region of 17,042 bp, and a pair of inverted repeats (IRs) of 25,648 bp. The genome contains 131 genes, including 83 protein-coding genes, 37 tRNA genes, and 8 rRNA genes. The GC contents in chloroplast genome, LSC region, SSC region, and IR region were 38.3%, 36.6%, 32.6%, and 43.1%, respectively. A total of 16 species are used to construct the phylogenetic tree of Paeoniaceae, the results showed that *P. rockii* subsp*. taibaishanica* is more closely related with congeneric *Paeonia suffruticosa* and *Paeonia ostii*, these species were clustered into a clade with high bootstrap support.

*Paeonia rockii* subsp. *taibaishanica* D. Y. Hong (Paeoniaceae), one of the tree peony species, is endemic to the Qinling Mountains in central China (Liu et al. 2019). The genus *Paeonia*, also known as the ‘King of Flowers’ in China, is an important source of traditional Chinese medicine (Bao et al. 2018). *P. rockii* subsp*. taibaishanica* have long been used to treat a range of cardiovascular and gynecological diseases (Liu et al. 2019). This species has been listed as endangered species in the IUCN Red List, and urgent management and conservation are required. In this study, we characterized the complete plastid genome sequence of *P. rockii* subsp. *taibaishanica* based on the Illumina pair-end sequencing data. The annotated chloroplast genome of *P. rockii* subsp. *taibaishanica* has been deposited into the GenBank with the accession number MW192444.

The fresh and healthy leaves of *P. rockii* subsp*. taibaishanica* were collected in Taibai county, Shaanxi Provincial National Nature Reserve (Baoji, China; N 34.062401, E107.700326; Alt.1564.41m). The dried tissue samples and voucher specimens (No. PRLZH2019022) were deposited in the Evolutionary Botany Group, Key Laboratory of Resource Biology and Biotechnology in Western China (Shaanxi, China). The total genomic DNA was extracted using the modified cetyl trimethyl ammonium bromide method (Doyle and Doyle 1987) and further assessed by agarose gel electrophoresis to check the quality of DNA. Then, the DNAs were subjected to Illumina sample preparation. We constructed a pair-end (PE) library with 350 bp insert size fragments using TruSeq DNA sample preparation kits (Sangon, Shanghai, China). The high throughput DNA molecular sequencing was indexed by the Illumina Hiseq 2500 technology platform. The obtained raw reads were trimmed using the program NGSQC Toolkit_version 2.3.3 with the default statistical genetic parameters (Patel and Jain 2012). After dislodged the low-quality DNA sequence reads, the clean DNA reads were assembled using the software MIRA version 4.0.2 (Chevreux et al. 2004) and MITObim version 1.8 (Hahn et al. 2013) with the complete plastid genome of congeneric *P. rockii* (NC037772) as the reference DNA sequences. The genome *P. rockii* subsp. *taibaishanica* was annotated by the programs PGA (Qu et al. 2019) and DOGAM (Wyman et al. 2014) (http://dogma.ccbb.utexas.edu/).

The complete plastid genome size of *P. rockii* subsp. *taibaishanica* is 153,368 bp in length, including a large single copy (LSC) region of 85,030 bp, a small single copy (SSC) region of 17,042 bp, and a pair of inverted repeats (IRs) of 25,648 bp. The genome contains 131 genes, including 83 protein-coding genes, 37 tRNA genes, and 8 rRNA genes. At the same time, the three genes *rpl22*, *rps18*, and *ycf1* were found to be pseudogenes. The GC content in plastid genome, LSC region, SSC region, and IR region were 38.3%, 36.6%, 32.6%, and 43.1%, respectively. Meanwhile, a total of 15 genes (*atpF*, *ndhA*, *ndhB*, *petB*, *petD*, *rpl2*, *rpl16*, *rps16*, *rpoC1*, *trnA-UGC*, *trnG-GCC*, *trnI-GAU*, *trnK-UUU*, *trnL-UAA*, and *trnV-UAC*) contained one intron, and three genes (*ycf3*, *rps12*, and *clpP*) contained two introns.

A total of 14 species from the genus *Paeonia* were used to construct the phylogenetic tree with two *Aconitum* species as outgroups. All of the 16 plastid sequences were aligned using the software MAFFT (Katoh and Standley 2013) with the default genetic parameters. The phylogenetic analysis was conducted using the program RAxML (Stamatakis 2006) with 1000 bootstrap replicates. The results showed that *P. rockii* subsp. *taibaishanica* was closely related to the two congeneric species *Paeonia suffruticosa* and *Paeonia ostii* (Figure 1).

**Figure 1.**
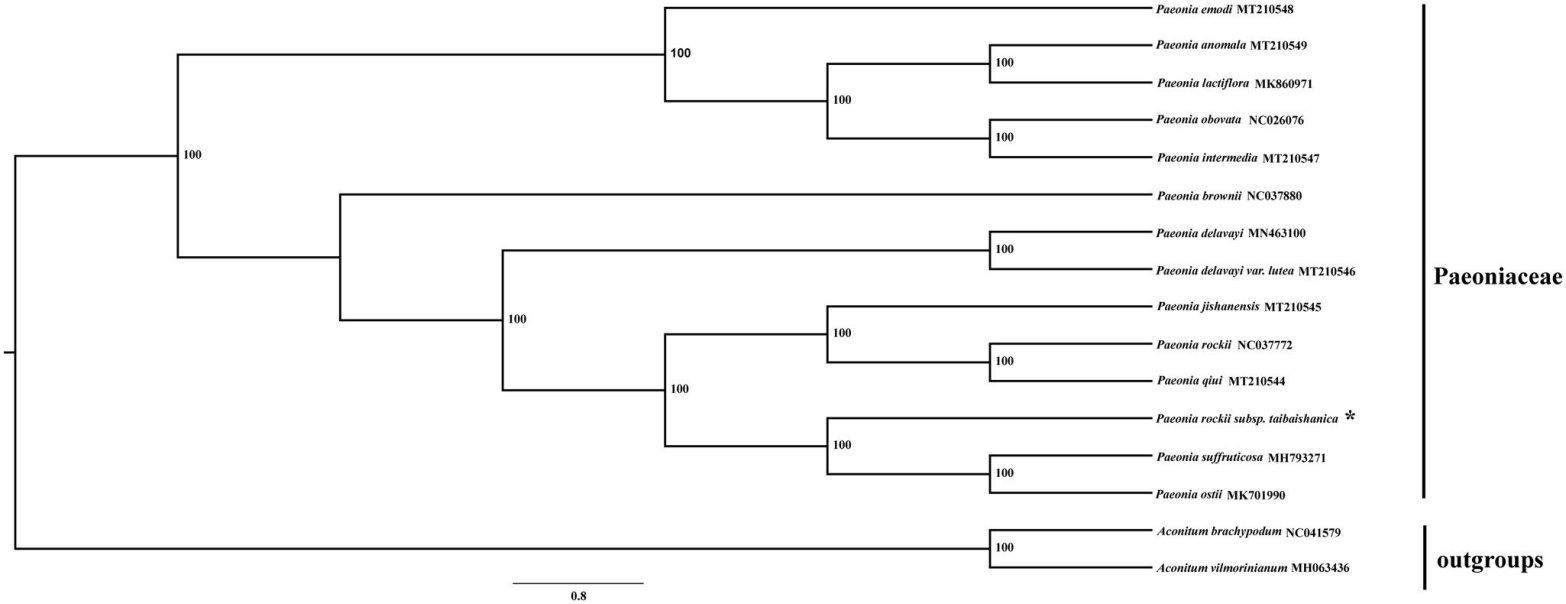
Phylogenetic relationship tree of *Paeonia rockii* subsp. *taibaishanica* and its related species based on the whole plastid genome sequences. *The newly obtained plastid genome of *Paeonia rockii* subsp. *taibaishanica*.

## Data Availability

The data that support the results of this work are openly available in GenBank at https://www.ncbi.nlm.nih.gov, reference number MW192444.
